# Acute and late toxicities of radiotherapy for patients with discoid lupus erythematosus: a retrospective case-control study

**DOI:** 10.1186/1748-717X-7-22

**Published:** 2012-02-16

**Authors:** Ajaykumar B Patel, Christopher L Hallemeier, Ivy A Petersen, Ashley W Jensen, Thomas G Osborn, Robert C Miller

**Affiliations:** 1 Department of Radiation Oncology, Mayo Clinic, 200 First St SW, Rochester, MN 55905, USA; 2 Division of Rheumatology, Mayo Clinic, Rochester, Minnesota, USA

**Keywords:** Connective tissue diseases, Discoid lupus erythematosus, Radiotherapy

## Abstract

**Background:**

The purpose of this study was to evaluate acute and late toxicities of radiotherapy for patients with discoid lupus erythematosus (DLE).

**Methods:**

A retrospective review was performed of patients with DLE who received radiotherapy at our institution between 1980 and 2005. Patients with other connective tissue disorders were excluded. Control patients were matched 2:1 with the DLE treatment courses based on age, cancer diagnosis, year of treatment, radiotherapy dose, and sex. Acute (within 30 days from the completion of radiotherapy) and late toxicities were evaluated for each treatment course using the Common Terminology Criteria for Adverse Events Version 3.0.

**Results:**

Twelve patients with DLE received a total of 15 radiotherapy courses. The median follow-up time was 2.6 years (range, 0.0-15.2 years). Acute toxicity of any organ was observed in 10 (67%) treatment courses, of which 2 (13%) were Grade 3 or higher. Acute Grade 1 or 2 dermatologic toxicity was observed in 8 courses (53%). Late toxicity of any organ was observed in 7 of 12 (58%) evaluable treatment courses, of which 3 (23%) were grade 3 or higher. Late grade 1 or 2 dermatologic toxicity was observed in 5 (42%) courses. No patient experienced acute or late Grade 3 or higher dermatologic toxicity. The rates of any organ or dermatologic acute and late toxicity were not significantly different between DLE and control treatment courses.

**Conclusions:**

Our findings do not suggest an increased risk of toxicity to the skin or other organs in patients with DLE receiving radiotherapy.

## Background

Discoid lupus erythematosus (DLE) is a subtype of cutaneous lupus erythematosus that is characterized by circular, red, patchy skin plaques (termed "discoid lesions"). These lesions usually appear on the scalp, cheeks, and nose but can also affect the neck, chest, back, and other areas of the head and body. Fifty percent of discoid lesions are found on hair-bearing scalp regions [1]. Diagnosis is differentiated from subacute cutaneous lupus because DLE can result in chronic scarring. Discoid lupus also is associated with increased photosensitivity, increased risk of sunburn, and discoid lesions exacerbated by sunlight. DLE is differentiated from systemic lupus erythematosus (SLE) in that patients with DLE have skin lesions only; whereas, patients with SLE have systemic features meeting the American College of Rheumatology SLE criteria. However, approximately 5-10% of patients diagnosed with DLE eventually develop SLE. The cause of DLE is unknown but may be due to an autoimmune disorder. It is a relatively rare disorder compared with SLE and generally affects more women than men. Diagnosis of DLE is confirmed with a skin biopsy. Total clearance of skin lesions can be achieved with early treatment consisting of potent topical corticosteroids and antimalarial agents; failure of treatment can lead to permanent scarring [1]. Newer therapies for DLE include pulsed dye laser treatment, phototherapy, and efalizumab (an anti-CD11a antibody) [2-4].

Many case reports in the literature have detailed unusual toxicity after radiotherapy in select patients with connective tissue disorders (CTD) such as rheumatoid arthritis, DLE, SLE, polymyositis, dermatomyositis, and scleroderma, but retrospective studies have reported more modest toxicity data in small groups of patients [5-7]. To our knowledge, no study has exclusively examined adverse effects of radiotherapy for patients with DLE. Because DLE is a disorder characterized by inflammatory attacks on the skin and by photosensitivity, it raises concerns about increased toxicity from radiotherapy.

This article is the third in a series of studies conducted at Mayo Clinic that assessed radiotherapy outcomes of cancer patients with CTD. The first and second analyses focused on systemic scleroderma and SLE, respectively [8,9]. The current study aimed to evaluate our institution's experience with late and acute toxicities of radiotherapy for patients with DLE.

## Methods

We queried the institutional master diagnosis database to identify patients with DLE treated at Mayo Clinic (Rochester, Minnesota) from January 1, 1980, through December 31, 2005. Records were cross-referenced against patient treatment records from the Department of Radiation Oncology to create the initial list of subjects with DLE who received radiotherapy. The diagnosis of DLE was confirmed by manually searching records for evidence of discoid rash. Patients with a confirmed diagnosis of SLE or with more than 4 American College of Rheumatology SLE criteria were included in a previous report on SLE [9] and excluded from the current investigation. Patients with other CTDs, including scleroderma and rheumatoid arthritis, also were excluded. Case controls were identified by searching the Department of Radiation Oncology database. Controls were matched 2:1 with the DLE treatment courses based on age, cancer diagnosis, year-of-treatment, dose, and sex.

Toxicities were evaluated using the Common Terminology Criteria for Adverse Events version 3.0 [10]. Toxic events were considered acute if they occurred during treatment or within 30 days after treatment completion. Events after 30 days were considered late toxicities. Each treatment course was evaluated for toxicities (some patients had more than 1 treatment). Survival rate and follow-up time were calculated from the radiotherapy completion date.

Patient characteristics and treatment characteristics were compared between the DLE patients and the controls using the 2-tail Fisher's exact test or one way analysis of variation (ANOVA), where appropriate. In the DLE patients, several patient and treatment characteristics were examined for an association with acute toxicity using the 2-tail Fisher's exact test or late toxicity using the log-rank test. Variables examined included age at radiotherapy (> 64 vs. ≤ 64), age at DLE diagnosis (> 59 vs. ≤ 59), surgical treatment (yes vs. no), chemotherapy administration (yes vs. no), patient sex (male vs. female), photosensitivity (presence vs. absence), timing of DLE diagnosis relative to initiation of radiotherapy (before vs. after), total radiotherapy dose (> 52.8 Gy vs. ≤ 52.8 Gy), dose per fraction (> 2 Gy vs. ≤ 2 Gy), treatment intent (curative vs. palliative), treatment site (breast, head and neck, pelvis, or thorax vs. other). Kaplan-Meier statistics were used to calculate late toxicity rates [11]. Acute and late toxicities rates were compared between the DLE treatment courses and the control treatment courses using the 2 tail Fisher's exact test and the log-rank test, respectively. A p-value of < 0.05 was considered significant. The statistical software used for analysis was JMP (SAS Institute Inc, Cary, North Carolina).

## Results

We identified 12 patients with a diagnosis of DLE who received a total of 15 courses of radiotherapy (1 patient received 2 courses and 1 patient received 3 courses). We identified 30 unique controls receiving 30 courses of radiotherapy. Patient and treatment characteristics for DLE patients and controls are detailed in Tables 1 and 2; there were no statistically significant differences between the two groups. For the DLE patients, 2 (17%) were alive and 10 (83%) had died (3 died of cancer) with a median follow-up time of 2.6 years (range, 0.0-15.2 years). For the control patients, 8 (27%) were alive and 28 (73%) had died (6 from cancer) with a median follow-up of 3.3 years (range 0.0-19.1 years).

**Table 1:** Patient Characteristics

Characteristic	DLE (n = 12)*	Controls (n = 30)
Female sex, No. of patients (%)	6 (50)	10 (33)

Race, white, No. of patients (%)	12 (100)	30 (100)

Age, median (range), y		

At radiotherapy	69 (55-82)	65 (51-86)

At initial DLE symptoms	47 (9-79)	-

At DLE diagnosis	45 (11-79)	-

DLE diagnosis		

Established before radiotherapy, No. of patients (%)	9 (75)	-

Established after radiotherapy, No. of patients (%)	3 (25)	--

Time between DLE diagnosis and radiotherapy, median (range), y		--

Diagnosis first	9.0 (2.2-27.0)	-

Radiotherapy first	3.1 (1.7-6.3)	-

American College of Rheumatology systemic lupus erythematosus characteristic, No. of patients (%)		

Discoid rash	12 (100)	-

Photosensitivity	9 (75)	-

Primary tumor location, No. of patients (%)		

Prostate	4 (33)	8 (26)

Breast	2 (17)	4 (13)

Lung	2 (17)	8 (26)

Oral	2 (16)	6 (20)

Anus	1 (8)	2 (6)

Bladder	1 (8)	2 (6)

Abbreviation: DLE, discoid lupus erythematosus

* 1 patient received 2 treatment courses and 1 patient received 3 treatment courses

**Table 2:** Treatment Course Characteristics

Characteristic	DLE (n = 15)	Controls (n = 30)
Intent of treatment, No. of treatments (%)		

Curative	12 (80)	24 (80)

Palliative	3 (20)	6 (20)

Anatomic target of radiotherapy, No. of treatments (%)		

Head and neck	3 (20)	6 (20)

Prostate	3 (20)	6 (20)

Breast	2 (13)	4 (13)

Thorax	2 (13)	4 (13)

Spine	2 (13)	4 (13)

Bladder	1 (7)	2 (7)

Brain	1 (7)	2 (7)

Pelvis	1 (7)	2 (7)

Radiotherapy technique, No. of treatments (%)		

External beam radiotherapy, 2-dimensional	13 (87)	28 (93)

External beam radiotherapy, 3-dimensional	1 (7)	0 (0)

Iodine brachytherapy	1 (7)	2 (7)

Radiotherapy dose, median (range)		

Dose per fraction, Gy	2.0 (1.2-8.0)	2.0 (1.6-4.0)

No. of fractions	28 (1-50)	30 (2-42))

Total dose, Gy	52.8 (8.0-66.0)	58.8 (8.0-66.6)

Biologically equivalent dose, Gy, median (range)		

Gy_3_	31.8 (18.2-45.5)	39.5 (24.3-45.5)

Gy_10_	19 (5.6-13.9)	12.3 (3.3-16.7)

Chemotherapy before or after 60 d of radiotherapy		

No	12 (80)	23 (76)

Yes	3 (20)	7 (24)

Surgery before or after 60 d of radiotherapy		

No	12 (80)	23 (76)

Yes	3 (20)	7 (24)

Abbreviation: DLE, discoid lupus erythematosus

### Acute toxicities

In the DLE patients, 10 of the 15 treatment courses (66%) were associated with any-grade acute toxicities. Two courses (13%) were associated with grade 3 or higher acute toxicities (grade 4 mucositis during tonsillar radiotherapy and grade 3 neck and back pain shortly after spine radiation). Eight courses (53%) were associated with grade 1 or 2 acute dermatologic toxicities (erythema in 6 courses, infection in 1 course, and pruritus in 1 course). No acute dermatologic toxicities higher than grade 2 were observed. All acute dermatologic toxicities subsided within 30 days. No treatment courses were interrupted because of acute toxicities. Univariate analysis did not show any statistically significant correlation between patient or treatment characteristics and any-grade, grade 3 or higher, or dermatologic acute toxicity.

In the control patients, 27 of the 30 treatment courses (90%) were associated with any-grade acute toxicities. One course (3%) was associated with grade 3 or higher acute toxicity (grade 4 pulmonary embolus). Eight courses (27%) were associated with grade 1 or 2 acute dermatologic toxicities. No acute dermatologic toxicities higher than grade 2 were observed.

Univariate analysis did not show a statistically significant difference in acute toxicity rates (any grade, grade 3 or higher, or dermatologic) between the DLE courses and the control courses.

### Late toxicities

**Figure 1:**
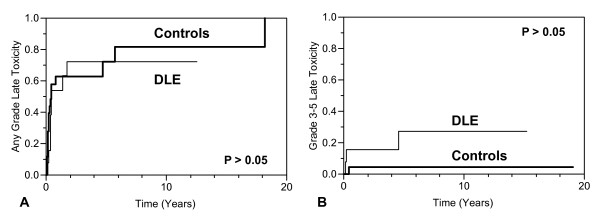
**Kaplan-Meier estimates of any grade (A) and grade 3-5 (B) late toxicity with DLE patient treatment courses (n = 13) and control patient treatment courses (n = 26)**. Log-Rank p-values were > 0.05 for both comparisons.

In the DLE patients, only 13 of the 15 courses were assessed for late toxicities (for 2 courses, no followup was available beyond 30 days). Seven of the 13 courses (54%) were associated with evidence of late toxicity. Three courses (23%) were associated with grade 3 late toxicity (low back pain and nocturia with 1 course, neck pain with 1 course, and conductive hearing loss with 1 course.) No grade 4 or 5 late toxicity was observed. Five courses (38%) were associated with late grade 1 or 2 late dermatologic toxicities (telangiectasia with 2 courses, alopecia with 1 course, actinic keratosis with 1 course, and unspecified perioral rash with 1 course). No late dermatologic toxicities higher than grade 2 were observed. The median time to any grade of late toxicity was 1.4 years (range, 0.3-12.5 years). The Kaplan-Meier estimates of all grades late toxicities at 2 years and 5 years were 72% for both (Figure 1A). The Kaplan-Meier estimates of grade 3 or higher late toxicities at 2 years and 5 years were 15% and 27%, respectively (Figure 1B). Univariate analysis did not show any statistically significant correlation between patient or treatment characteristics and any-grade, grade 3 or higher, or dermatologic late toxicity.

In the controls, only 26 of the 30 treatment courses were evaluated for late toxicities (for 4 courses, no followup was available beyond 30 days). Eight out of 26 courses (31%) were associated with late toxicity of any grade. One course (4%) was associated with grade 3 late toxicity. No grade 4 or 5 late toxicities were observed. Seven courses (27%) were associated with grade 1 or 2 late dermatologic toxicity. No late dermatologic toxicities higher than grade 2 were observed.

Univariate analysis did not show a statistically significant difference in late toxicity rates (any grade, grade 3 or higher, or dermatologic) between the DLE courses and the control courses.

## Discussion

In this retrospective study of patients with DLE who received radiotherapy at our institution, severe (grade 3+) acute or late toxicity was infrequent. No acute or late dermatologic toxicity greater than grade 2 was observed. The incidence of acute and late toxicity in DLE patients was similar to that of matched controls. Thus, our data suggest that radiotherapy can be administered safely in patients with DLE.

CTDs such as DLE and SLE have long been thought to increase the risk of radiotherapy-related toxicities [12]. Concerns stem from the possibility of pathologic damage to microvasculature, which could lead to fibrosis, and other effects. The hypothesis that patients with CTDs potentially have increased radiosensitivity is supported by numerous case reports that have described exaggerated complications after radiotherapy in patients with collagen vascular diseases [13-15]. Nevertheless, larger, retrospective studies [8,9,12,16-18] and a pooled data analysis from Holscher et al. [6] have suggested an increased, but less dramatic, risk of higher toxicity in patients with CTD who receive radiotherapy. The largest study to date of patients with CTD was conducted by Morris and Powell [16]; they included 209 patients with various collagen vascular diseases who received radiotherapy. The authors concluded that patients with non-rheumatoid arthritis CTDs had an increased risk of late toxicities with radiotherapy and that treatment decisions should be made on a case-by-case basis for radiosensitive patients. Research at Mayo Clinic has suggested that risk of late complications after radiotherapy increases with the increasing severity of the underlying CTD [19].

Concerns about toxicity have led to a marked reduction in radiotherapy for patients with CTDs. A study by Benk et al. [20] showed that only 10% of patients with SLE referred for radiotherapy received treatment, although 65% of patients without CTDs typically are treated. They also showed that the patients receiving treatment did not have any unusual toxicity. The authors concluded that radiotherapy may be inappropriately withheld from patients with SLE and cancer.

Several case reports have noted an increased risk of toxicity in patients with DLE [5,21,22]. De Naeyer et al. [5] reviewed the literature on patients with CTD receiving radiotherapy and described a single patient who had 2 courses of radiotherapy, one before the onset of DLE and another 3 years later. The patient experienced severe acute and late toxicity only after the second treatment course. Eedy and Corbett [21] described a patient who received radiotherapy to the chest 10 months after a diagnosis of DLE. Within 10 days of treatment, characteristic discoid lesions developed in the precise region of the radiation field. Treatment with topical corticosteroids and chloroquine resolved the lesions. His original erythematous regions on the cheeks and nose remained, and no other toxicities were noted. Ishida-Yamamoto et al. [22] detailed a patient with a discoid lesion exacerbated by radiotherapy for lymphoma. A biopsy diagnosis of DLE was made when the nodule grew larger. Treatment with a topical corticosteroid resulted in dramatic improvement and left only a scar. The patient had no episodes of DLE recurrence in 4 years of follow-up.

In contrast to these case reports, no patient in our study had acute or late dermatologic toxicities higher than grade 2. None of the patients in our study had clinically significant exacerbation or development of discoid lesions with radiotherapy. Importantly, we note that none of the patients had clear evidence of discoid lesions in the radiation field before therapy. We did note that the risk of grade 3 or higher late toxicity was numerically higher in the DLE group compared to the control group, although this difference was not statistically significant. The small sample size in the present study limits the ability to exclude a small but statistically significant difference in late toxicity. However, the observed late grade 3 toxicities were interpreted as expected toxicities of radiation unrelated to DLE. Additionally, it is reassuring that there were no late grade 4-5 toxicities observed.

There are limitations to the conclusions that can be drawn from our current investigation. This was a retrospective chart review, thus unappreciated biases may have been present. The sample size was small, thus limiting the ability to identify potential patient and/or treatment factors associated with increased toxicity. The patient population and radiotherapy treatments administered were heterogeneous. No patient in our series had documented discoid lesions in the radiotherapy treatment field, thus the safety of administering radiotherapy in this setting remains undefined. In spite of these limitations, this is to our knowledge the largest series of patients with DLE and radiotherapy reported to date in the medical literature. Further studies are needed to confirm or refute the safety of radiotherapy in patients with DLE and to identify potential prognostic factors that may predict for increased toxicity.

## Conclusions

In our cohort of patients with DLE, severe dermatologic toxicity from radiotherapy was not observed. DLE should not be considered an absolute contraindication to radiotherapy. However, previous case reports suggest that precautions should be taken if discoid lesions are present in the radiation field. Although previous studies suggest a modest increase in the risk of late toxicity with radiotherapy in the setting of CTDs, the current study found no evidence supporting a prohibitive increase in the incidence of grade 3 or higher late toxicities after radiotherapy in patients with DLE. Decisions on radiotherapy in patients with CTD should be made on an individual basis and consider the risks posed by the malignant disease.

## Competing interests

The authors declare that they have no competing interests.

## Authors' contributions

All authors were involved in the study conception and design. ABP and RCM acquired the data. ABP, CLH, and RCM performed the data analysis and interpretation. ABP and CLH were the primary authors for the manuscript. All authors were involved in revising the manuscript. All authors approved the final manuscript.

## References

[B1] Panjwani S Early diagnosis and treatment of discoid lupus erythematosus J Am Board Fam Med 2009 22 2 206 213 10.3122/jabfm.2009.02.080075 19264946

[B2] Erceg A Bovenschen HJ van de Kerkhof PC de Jong EM Seyger MM Efficacy and safety of pulsed dye laser treatment for cutaneous discoid lupus erythematosus J Am Acad Dermatol 2009 60 4 626 632 10.1016/j.jaad.2008.11.904 19293010

[B3] Booken N Schumann T Fuchslocher M Goerdt S Goebeler M Successful therapy of discoid lupus erythematosus with efalizumab Hautarzt 2010 61 3 246 249 German 10.1007/s00105-009-1767-4 19436973

[B4] Mitra A Yung A Goulden V Goodfield MD A trial of low-dose UVA1 phototherapy for two patients with recalcitrant discoid lupus erythematosus Clin Exp Dermatol 2006 31 2 299 300 10.1111/j.1365-2230.2005.02030.x 16487126

[B5] De Naeyer B De Meerleer G Braems S Vakaet L Huys J Collagen vascular diseases and radiation therapy: a critical review Int J Radiat Oncol Biol Phys 1999 44 5 975 980 10.1016/S0360-3016(99)00103-0 10421528

[B6] Holscher T Bentzen SM Baumann M Influence of connective tissue diseases on the expression of radiation side effects: a systematic review Radiother Oncol 2006 78 2 123 130 10.1016/j.radonc.2005.12.013 16445999

[B7] Lin A Abu-Isa E Griffith KA Ben-Josef E Toxicity of radiotherapy in patients with collagen vascular disease Cancer 2008 113 3 648 653 10.1002/cncr.23591 18506734

[B8] Gold DG Miller RC Petersen IA Osborn TG Radiotherapy for malignancy in patients with scleroderma: the Mayo Clinic experience Int J Radiat Oncol Biol Phys 2007 67 2 559 67 10.1016/j.ijrobp.2006.09.003 17236971

[B9] Pinn ME Gold DG Petersen IA Osborn TG Brown PD Miller RC Systemic lupus erythematosus, radiotherapy, and the risk of acute and chronic toxicity: the Mayo Clinic Experience Int J Radiat Oncol Biol Phys 2008 71 2 498 506 10.1016/j.ijrobp.2007.10.014 18164848

[B10] 2006 Cancer Therapy Evaluation Program, Common Terminology Criteria for Adverse Events, Version 3.0, DCTD, NCI, NIH, DHHS March 31, 2003 http://ctep.cancer.gov

[B11] Kaplan E Meier P Nonparametric estimation from incomplete observations J Am Stat Assoc 1958 53 5 457 481

[B12] Ross JG Hussey DH Mayr NA Davis CS Acute and late reactions to radiation therapy in patients with collagen vascular diseases Cancer 1993 71 11 3744 3752 10.1002/1097-0142(19930601)71:11<3744::AID-CNCR2820711144>3.0.CO;2-C 8490925

[B13] Olivotto IA Fairey RN Gillies JH Stein H Fatal outcome of pelvic radiotherapy for carcinoma of the cervix in a patient with systemic lupus erythematosis Clin Radiol 1989 40 1 83 84 10.1016/S0009-9260(89)80040-6 2920524

[B14] Robertson JM Clarke DH Pevzner MM Matter RC Breast conservation therapy. Severe breast fibrosis after radiation therapy in patients with collagen vascular disease Cancer 1991 68 3 502 508 10.1002/1097-0142(19910801)68:3<502::AID-CNCR2820680310>3.0.CO;2-V 1648431

[B15] Fleck R McNeese MD Ellerbroek NA Hunter TA Holmes FA Consequences of breast irradiation in patients with pre-existing collagen vascular diseases Int J Radiat Oncol Biol Phys 1989 17 4 829 833 10.1016/0360-3016(89)90074-6 2777673

[B16] Morris MM Powell SN Irradiation in the setting of collagen vascular disease: acute and late complications J Clin Oncol 1997 15 7 2728 2735 9215847 10.1200/JCO.1997.15.7.2728

[B17] Phan C Mindrum M Silverman C Paris K Spanos W Matched-control retrospective study of the acute and late complications in patients with collagen vascular diseases treated with radiation therapy Cancer J 2003 9 6 461 466 10.1097/00130404-200311000-00005 14740974

[B18] Chen AM Obedian E Haffty BG Breast-conserving therapy in the setting of collagen vascular disease Cancer J 2001 7 6 480 491 11769860

[B19] Gold DG Miller RC Pinn ME Osborn TG Petersen IA Brown PD Chronic toxicity risk after radiotherapy for patients with systemic sclerosis (systemic scleroderma) or systemic lupus erythematosus: association with connective tissue disorder severity Radiother Oncol 2008 87 1 127 131 10.1016/j.radonc.2007.11.031 18158195

[B20] Benk V Al-Herz A Gladman D Urowitz M Fortin PR Role of radiation therapy in patients with a diagnosis of both systemic lupus erythematosus and cancer Arthritis Rheum 2005 53 1 67 72 10.1002/art.20912 15696566

[B21] Eedy DJ Corbett JR Discoid lupus erythematosus exacerbated by x-ray irradiation Clin Exp Dermatol 1988 13 3 202 203 10.1111/j.1365-2230.1988.tb01972.x 3246083

[B22] Ishida-Yamamoto A Kanno K Sato E Takahashi H Iizuka H Discoid lupus erythematosus exacerbated by radiation therapy J Dermatol 2005 32 3 225 226 15863874 10.1111/j.1346-8138.2005.tb00752.x

